# Prevalence of hearing loss and use of hearing aids among children and adolescents in Germany: a systematic review

**DOI:** 10.1186/s12889-019-7602-7

**Published:** 2019-09-18

**Authors:** C. Schmucker, P. Kapp, E. Motschall, J. Loehler, J. J. Meerpohl

**Affiliations:** 1 Institute for Evidence in Medicine, Medical Center - University of Freiburg, Faculty of Medicine, University of Freiburg, Freiburg, Germany; 2 Faculty of Medicine and Medical Center, Institute of Medical Biometry and Statistics, University of Freiburg, Freiburg, Germany; 3 Department of Otorhinolaryngology, Head and Neck Surgery, University Hospital of Schleswig-Holstein, Lübeck, Germany; 4 German Study Centre for Otorhinolaryngology, Head and Neck Surgery (DSZ - HNO), Bonn, Germany

**Keywords:** Hearing loss, Prevalence, children, adolescents, Systematic review, Germany

## Abstract

**Background:**

Current data suggest that approximately 466 million people (5.0%) of the world’s population have disabling hearing loss, therefrom, 34 million children, impacting their quality of life. To provide estimates on the prevalence of hearing loss on a national level, we reviewed the epidemiological literature addressing hearing loss in children and adolescents living in Germany as an example for a Western country.

**Methods:**

We searched Medline, Web of Science, Cochrane Library, ScienceDirect and LIVIVO to identify published data. Furthermore, we manually searched websites of relevant institutions and journals not listed in electronically and searched for ongoing studies and/or not yet published data in clinicaltrials.gov. Study selection, data extraction, and methodological assessment were carried out by two reviewers.

**Results:**

In total, 11 reports provided data with sample sizes ranging from 310 up to more than 14 million children and adolescents. Prevalence data were collected by interviews (self-assessments), using pure-tone audiometry or the international classification of diseases (ICD-10) coding and ranged from 0.1 to 128 per 1000 children. Although the estimate of the prevalence of hearing loss goes down, when the threshold was raised, generating a comprehensive and coherent set of estimates proved challenging owing to clinical heterogeneity including variation in age, the study setting, the definition of hearing loss and the assessment method. Moreover, representativeness (external validity) was often impaired owing to estimates lacking currentness (i.e., referring to former West Germany) or selected (patient) data and may not be typical for a more general population.

**Conclusions:**

In conclusions, this work raises public awareness of the high prevalence of hearing loss, highlights issues associated with epidemiological research and is of great importance for researcher and those who use epidemiological data to inform clinical and political decision making.

## Background

Hearing loss is the fourth highest cause of disability globally [1]. Current data suggest that approximately 5% of the world’s population —32 million adults and 34 million children and adolescents— suffer from disabling hearing loss, defined as hearing loss greater than 40 dB hearing levels (dB HL) in the better hearing ear in adults and greater than 30 dB HL in the better hearing ear in children [2–4]. According to the World Health Organisation (WHO) adverse impacts of unaddressed (untreated) disabling hearing loss, cause annual global costs of over 660 billion Euros [5]. These overall costs include expenses associated with the health-care and education systems (direct costs), costs including productivity losses due to absenteeism from work as well as income loss by family members caring for a disabled child (indirect costs) and costs for accessibility, adaptation and social inclusion for people with disabilities (intangible/societal costs) [5]. Within the European Union, approximately 22.6 million people live with such an untreated, disabling hearing loss leading to annual overall costs of 185 billion Euros or 8200 Euros per affected person [3].

Overall, it is assumed that half of all cases of hearing loss in children could be prevented through public health measures. Particularly, early detection (e.g. by newborn, infant, pre-school and/or school hearing screening programs) and therapeutic management are crucial to minimize the impact of hearing loss on a child’s development (including social isolation, psychological problems and educational achievements) and prospects for personal growth later in life [6–8]. Those affected can benefit from the use of hearing devices, such as hearing aids, cochlear implants, and other assistive devices. They may also benefit from speech therapy, aural rehabilitation or related services [9]. However, only 15 to 30% of those requiring treatment are receiving adequate management [10].

Data on the prevalence and incidence of hearing loss are, however, often difficult to identify, outdated, or may not reflect the current population. This work aimed to estimate the number of children and adolescents with hearing loss and the proportion of children wearing hearing aids using available data from the general population living in Germany as an example for a Western country. Considering the methodological quality (risk of bias and representativeness) from these prevalence data this approach allows us to highlight potential issues associated with epidemiological research and judge whether there is sufficient evidence-based knowledge in this otologic research area on a national level [11]. This systematic review also complements a preceding systematic review providing estimates for the adult German population [12]. This set of systematic reviews is part of the ongoing effort of the German Study Centre for Otorhinolaryngology, established by the German Society of Otorhinolaryngology, Head, and Neck Surgery and the German Professional Association of Ear-Nose- and Throat Surgeons, to improve evidence-based research planning and inform clinical and political decision making [11].

## Methods

The present systematic review was planned and conducted according to rigorous methodological standards, and reported in adherence to the PRISMA statement [13]. An a priori developed review protocol is available from the corresponding author. The methodology for the literature search, data extraction and bias assessment has been published previously [12].

### Inclusion criteria and literature search

We included published studies and/or other data sources providing estimates on the prevalence (frequency of the disorder, i.e., the proportion of cases) and/or incidence (number of new cases in a defined observation period) of hearing disorder in children and adolescents (up to 19 years of age) living in Germany. Studies addressing exclusively newborns or populations with specific diseases such as children with genetic defects or metabolic disorders were excluded as well as studies conducted prior to 1975 (we decided to use the cut-off year 1975 owing to the very long time lapse and associated demographical, socio-economical and medical-technical changes since then). No restrictions were made in relation to the design of the included studies.

A systematic literature search for studies published in German or English language was carried out in May 2017 and an update search was conducted in March 2019. We searched Medline, Medline Daily Update, Medline In Process, and other Non-Indexed Citations (Ovid), Web of Science (Clarivate Analytics), Cochrane Library (www.cochranelibrary.com), ScienceDirect (Elsevier), and LIVIVO. Additionally, we searched the bibliographies of relevant studies to identify further citations. A search was also conducted for ongoing or completed but not yet published studies in clinicaltrials.gov and the German study register (www.drks.de). Furthermore, the websites of different national institutes gathering epidemiological data were searched (e.g., the Robert-Koch-Institute (www.rki.de), the National Association of Statutory Health Insurance Physicians (www.kbv.de), and the German Federal Statistics Office (www.destatis.de)). The search strategy used in Medline (Ovid) is presented in the Additional file 1.

### Study selection

Two reviewers (PK and CS) screened the titles and abstracts of all reports identified by the searches. Thereafter, full-text copies of all potentially relevant articles were obtained and were assessed for eligibility.

### Data extraction

The following data were extracted independently by the two reviewers mentioned above: *(i)* key study characteristics (bibliographical data, study design, geographical area where data were collected, period of data collection, age and number of included children and/or adolescents, and both the definition and assessment method of the hearing loss); *(ii)* prevalence data and / or incidence of hearing loss (stratified by assessment method, age and severity [definition of hearing loss]) as well as information on the proportion of these children with hearing aids or cochlear implants.

### Risk of bias and data representativeness

The risk of bias and the representativeness was assessed considering pre-defined criteria which were developed by our group based on other epidemiological research [14]. Thereby, risk of bias assessment was based on: *(i)* the validity of data collection, i.e., whether the prevalence of hearing loss was judged by the respondents themselves (e.g. in an interview; high risk of bias), or whether the studies applied standardized procedures (e.g. pure-tone audiogram; low risk of bias); *(ii)* specification of the hearing loss, i.e., whether the hearing loss was defined after standardized criteria (e.g. in accordance with WHO criteria; low risk of bias) or whether no adequate definition was used (this refers primarily to self-reported hearing loss; high risk of bias); *(iii)* the completeness of data, i.e., whether all recruited children (whole study sample) were considered when data were analyzed (low risk of bias) or whether data were missing (e.g. due to drop-outs; high risk of bias). *Data representativeness* based on the characteristics of the study sample; i.e., when a selected sample (e.g. children and adolescents from one region or city in Germany) was considered to derive prevalence estimates, representativeness was judged as “low”, whereas data representativeness was judged as “high” when the study included a broad-ranging sample reflecting the entire adolescent population living in Germany.

Of note, both for data extraction and the methodological assessments, we relied on information provided in the individual study reports. If no judgment could be made owing to missing information (poor reporting), the corresponding item for risk of bias or data representativeness was classified as “unclear”.

## Results

### Systematic literature search

The systematic literature search identified 2601 references. Additionally, we identified 63 references by hand searching, including 31 registry entries referring to ongoing or completed and not yet published studies (clinicalstrials.gov [*n* = 12] and the DRKS register [*n* = 19]). In total, 2113 references were excluded by title and/or abstract screening because they did not address our research question.

**Fig. 1:**
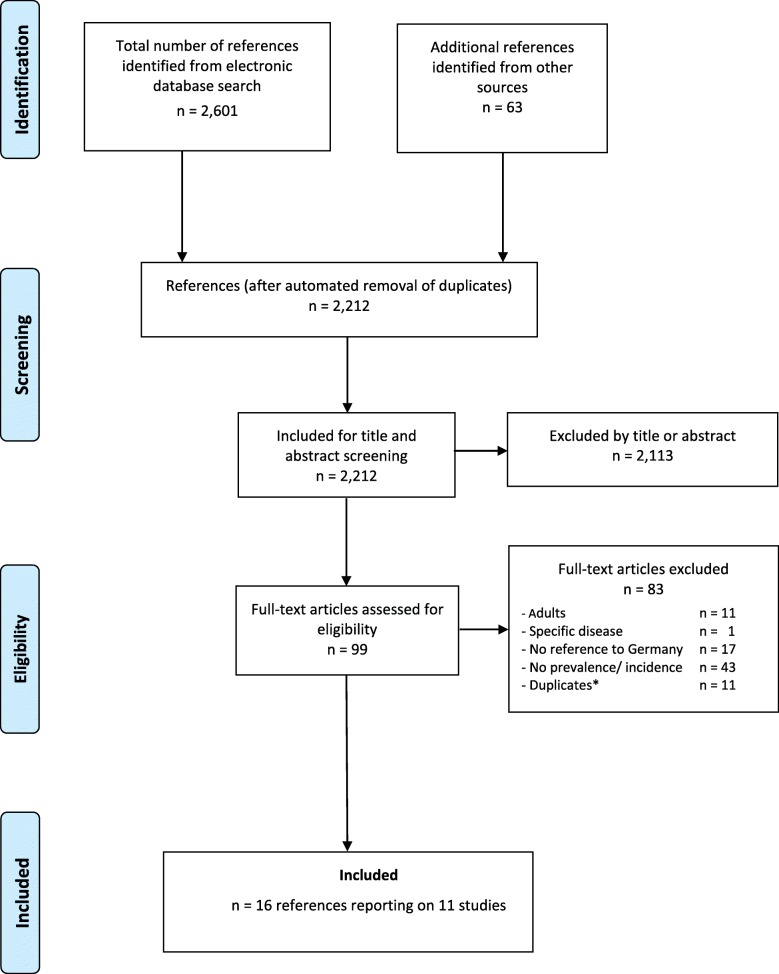
Flow chart of the literature search and study inclusion

Finally, 99 potentially relevant references were included for full-text screening. From these, 11 studies (reported in 16 references) provided data on the prevalence of hearing disorders in children and adolescents. The study flow diagram is presented in Fig. 1 (PRISMA flowchart) [13].

### Study characteristics

Table 1 outlines the study key characteristics. In short, data collection took place between 1977 [29, 30] and 2015 [21] and sample sizes ranged from 310 [17], over several and multiple tens of thousands, up to more than 14 million [21]. The study population included preschool children (between four and seven years of age) [19, 20], school children (up to the age of 14 years) [15, 18, 29, 30], youngsters (between 15 and 19 years of age) [16, 17], or a wide age range covering infants and adolescents up to 19 years of age [21–28]. The majority of studies gathered prevalence data throughout Germany [15–18, 21–25, 29, 30], one throughout the federal state Baden-Wuerttemberg [19], one within the city Cologne (and surroundings) [26–28] and four studies were restricted to the western German states (because data collection took place before 1990) [17, 20, 29, 30]. Children and adolescents with hearing loss were identified by (i) surveys/interviews (i.e., self-reported hearing loss [15–17]), (ii) (pre-)school hearing screening programs [18–20], (iii) using data collected in national registries or special facilities for hearing impaired children [21–30].

**Table 1:** Study key characteristics and findings

Studies	Study design	Year of data collection	Geographical region	Population			Definition of hearing loss	Prevalence (%)
				Setting (including study sample)	Age (years)	N		
Studies based on self-reported hearing loss by child or caregiver (using a questionnaire)								
EuroTrak^a^ 2017 [15]	cross-sectional (prospective)	2015	Germany	“balanced” sample (in respect to age, sex and geographical region)	≤14	1323	any hearing impairment/loss	3.1
WaBoLu 1993 [16]	cross-sectional (prospective)	1991	Germany	random sample (residents’ registration office)	18–19	505	any hearing impairment/loss	1.0 “no differences in gender and academic levels”
Stange 1992 [17]	cross-sectional (prospective)	1985	Former West Germany	random sample (households)	15–19	310	any hearing impairment/loss	4.0
Studies reporting hearing loss measured in screening programs								
KiGGS 2009 [18]	cross-sectional (prospective)	2003–2006	Germany	random sample (school children, residents’ registration office)	8–14	959	> 20 dB HL (1–6 kHz) uni- and/or bilateral	12.8
							> 30 dB HL (1–6 kHz) uni- and/or bilateral	2.4
RKI 2006 [19]	cross-sectional (retrospective)	1991–2002	Baden-Württemberg	preschool children participating in a screening program	4–5	1991: 96,641^b^	> 30 dB HL (0.5–6.0 kHz) uni- and/or bilateral	3.9
						1992: 104,615		3.9
						1993: 106,000		3.9
						1994: 105,150		4.0
						1995: 106,805		4.1
						1996: 108,063		4.8
						1997: 100,063		4.8
						1998: 109,391		4.6
						1999: 101,590		4.4
						2000: 98,822		4.5
						2001: 100,058		5.2
						2002: 103,489		4.7
Kruppa 1995 [20]	cross-sectional (prospective)	1988	Former West Germany	preschool children participating in a screening program	6–7	2032	> 20 dB HL (0.5–5.0 kHz) uni- and/or bilateral	7.4
Studies reporting hearing loss based on data collected in registries								
Destatis 2015 [21]	cross-sectional (retrospective)	2015	Germany	**source population:** children/adolescents living in Germany **cases:** data from the disability statistics	0–18	14,198,848	deafness (not further defined)	0.01
Neubauer 2011 [22]	cross-sectional (retrospective)	2010	Germany	**source population:** children/adolescents living in Germany **cases:** patient data/ICD coding transmitted for invoicing	0–18	nr	ICD coding^c^ uni- and/or bilateral	4.0
DZH 2003 [23–25]	cross-sectional (retrospective)	1996–2000	Germany	**source population:** children living in Germany **cases:** patient data / diagnoses of selected ENT doctors transferred data to the DZH^d^	nr	nr	permanent hearing loss (not further defined) uni- and/or bilateral	0.12
Streppel 2000 [26–28]	cross-sectional (retrospective)	1992–1993 [28]	Cologne and surroundings	**source population:** children/adolescents living in Cologne and surroundings **cases:** impaired children/adolescents attending special institutes i	2–19	738,500	≥40 dB HL (0.5–4.0 kHz) bilateral	0.04 (% males: 53.5)
							moderate: 40–70 dB HL	12.1
							severe: 70–95 dB HL	20.7
							profound: 95 dB HL	67.2
CRM 1982 [29, 30]	cross-sectional (retrospective)	1977	Former West Germany	**source population:** children born in 1969 **cases:** not clear which registry was used to identify cases	8	903,456	> 50 dB HL (0.5–2.0 kHz) uni- and/or bilateral	0.08 (% males: 56.1)

*CRM* Committee on Medical and Public Health Research; *dB HL* decibel hearing level; *Destatis* Federal Statistical Office of Germany; *DZH* German Registry for Hearing Loss in Children; *ICD* International classification of diseases; *kHz* Kilohertz; *KiGGS* German Health Interview and Examination Survey for Children and Adolescents; *RKI* Robert-Koch-Institute (German Public Health Institute); *WaBoLu* Institute for Water-, Ground- and Air-Hygiene; *PTA* Pure Tone Audiometry

^**a**^Since 2009 the European Hearing Instrument Manufacturers Association (EHIMA; www.ehima.com) has carried out surveys approximately every three years to determine hearing status and hearing aid usage in Europe over different age groups. The surveys are carried out by questionnaires and are designed to be comparable with the MarkeTrak surveys carried out in the USA

^**b**^Numbers available at: https://www-genesis.destatis.de/genesis/online/link/tabellen/12411

^**c**^ICD-10: H 83.3: noise effects on the inner ear; H 83.8: other specified diseases on the inner ear; H 83.9: disease of the inner ear, unspecified; H 90–H 90.8: conductive and sensorineural hearing loss; H 91.0–H 91.9: other hearing loss

^**d**^Mainly data from clinics, but also from outpatient facilities (112 institutions)

### Prevalence of hearing loss

Data on the prevalence of hearing loss - stratified (where possible) by assessment method, age, and definition of hearing loss - are provided in Table 1. Except for one study providing a separate estimate for bilateral hearing loss [26–28], the published estimates refer to uni- and/or bilateral hearing loss.

*Self-reported hearing loss:* prevalence of any self-reported hearing loss in adolescents up to 19 years of age ranged from 1.0% (23) to 4.0% (18). *Data from screening programs:* prevalence estimates based on preschool screening programs (> 30 dB HL) (conducted between 1991 and 2002) ranged from 3.9 to 5.2% (19). Using a threshold of > 20 dB HL impairment, the prevalence estimate in preschool children increased up to 7.4% (20). In comparison, a nationwide screening study estimated a prevalence of 2.4% (> 30 dB HL) and 12.8% (> 20 dB HL) in school children [18]. *Data from registries or special facilities:* Using the most current data from the German disability registry from 2015, the prevalence of severe hearing loss (defined as “deafness” without providing any threshold) –across all age groups up to 18 years of age- was reported to be 0.01% (17). This estimate is slightly lower than one that was recorded in 1977 suggesting that 0.08% of children at eight years of age show a hearing loss (> 50 dB HL) [29, 30]. In the 1990s, bilateral hearing loss (> 40 dB HL) was estimated to be 0.04% on the basis of children and adolescents attending special institutes [26–28], whereas data from the German study registry suggested a prevalence of permanent hearing loss of 0.12% (without providing any hearing threshold [23–25]). Using the international classification of diseases (ICD-10) coding the prevalence of hearing loss was estimated to be 4% (24). Overall, two studies reported a slightly higher proportion of hearing loss in males than in females (ratio males/females was 1.23 [26–28]).

### Prevalence of hearing aids

Only one study including children and adolescents of 14 years and younger provided self-reported data on the prevalence of hearing aids [15]. Hearing aid use in those with hearing loss was 32%. We did not identify any study reporting data on the prevalence of implantable hearing devices or cochlear implants in the general population.

### Assessment of risk of bias and representativeness

The respective assessments are shown in Table 2. Owing to a lacking objective case ascertainment, the studies based on self-reported hearing loss [15–17] or ICD-10 coding (including a variety of reasons/indications for consulting a physician, not only hearing loss) [22] were considered to be of high risk of bias. When standardized audiometric testing and hence objective thresholds for determining hearing loss were used –which was the case in all screening studies and two studies providing estimates based on national registries– risk of bias was judged to be low [18–20, 26–30]. All but two studies (four references [22–25]) provided the number of the full study sample (source population) allowing a verification of the provided estimates.

**Table 2:** Assessment of risk of bias and representativeness

Studies	Risk of Bias			Representativeness
	Was hearing loss measured in a valid way (validity of data collection [assessment method])?	Was hearing loss defined adequately (e.g., in accordance with WHO criteria)?	Are data for the full sample available and used for estimation of prevalence?	Is the data representative for the general population (children and adolescents) living in Germany?
Studies based on self-reported hearing loss by child or caregiver				
EuroTrak 2017 [15]	high RoB^a^	high RoB^a^	low RoB	unclear representativeness^b^
WaBoLu 1993 [16]	high RoB^a^	high RoB^a^	low RoB	high representativeness
Stange 1992 [17]	high RoB^a^	high RoB^a^	low RoB	lacking representativeness^c^
Studies reporting hearing loss measured in screening programs				
KiGGS 2009 [18]	low RoB	low RoB	low RoB	high representativeness
RKI 2006 [19]	low RoB	low RoB	low RoB	lacking representativeness^d^
Kruppa 1995 [20]	low RoB	low RoB	low RoB	lacking representativeness^c^
Studies reporting hearing loss based on data collected in registries				
Destatis 2015 [21]	low RoB	unclear RoB^e^	low RoB	high representativeness
Neubauer 2011 [22]	high RoB^f^	high RoB^f^	unclear RoB^g^	lacking representativeness^h^
DZH 2003 [23–25]	low RoB	unclear RoB^e^	unclear RoB^g^	lacking representativeness^h^
Streppel 2000 [26–28]	low RoB	low RoB	low RoB	lacking representativeness^d^
CRM 1982 [29, 30]	low RoB	low RoB	low RoB	lacking representativeness^c^

*CRM* Committee on Medical and Public Health Research; *Destatis* Federal Statistical Office of Germany; *DZH* German Registry for Hearing Loss in Children; *KiGGS* German Health Interview and Examination Survey for Children and Adolescents; *Robert-Koch-Institute (German Public Health Institute); RoB* Risk of Bias; *WaBoLu* Institute for Water-, Ground- and Air-Hygiene; *PTA* Pure Tone Audiometry

^a^No objective case ascertainment (subjective self-assessment) resulting in high risk of bias

^b^ Owing to missing information (poor reporting) data representativeness could not be ascertained resulting in unclear representativeness

^c^Not up-to-date, data collection before 1990 (data refer to the former West Germany) resulting in lacking representativeness

^d^Study sample is based on children and adolescents living in a special region (either in the federal state Baden Wuerttemberg [19] or in the city/area of Cologne [26–28]) resulting in lacking representativeness

^e^Owing to missing information (poor reporting), bias due to applying no standardized criteria (i.e., not applying WHO criteria) to define hearing loss cannot be fully excluded resulting in unclear risk of bias

^f^Case ascertainment was based on international classification of diseases (ICD) coding including a variety of reasons for consulting physicians (i.e., not only hearing loss data were collected in this registry) resulting in high risk of bias

^g^Owing to missing information (poor reporting), bias due to incomplete data cannot be fully excluded; the studies did not provide the number of the full study sample (source population) resulting in unclear risk of bias

^h^Only selected clinicans transferred data to the German registry for hearing loss in children, limiting the representativeness to the entire population living in Germany [23–25] and/or data from patients seeking medical advice rather than the general population were collected [22–25] resulting in lacking representativeness

Data representativeness (external validity) was an issue in most studies: (i) the data collected in registries were provided only by selected physicians [23–25] and/or based on patients seeking medical advice rather than the general population [22–25]; (ii) the study sample referred to children and adolescents living in the old West German states and were, therefore, out of date [17, 20, 29, 30]; or (iii) the study sample was based on children and adolescents living in a special region (city/area of Cologne [26–28] or in the federal state Baden-Wuerttemberg [19]) limiting the representativeness of the entire population living in Germany.

Overall, two studies were judged to be at low risk of bias and of high representativeness [18, 21].

## Discussion

The prevalence of hearing loss in the child and adolescent German population ranged from 0.1 to 128 per 1000 children and adolescents. Even though the prevalence of hearing loss decreases, when the hearing threshold is raised (e.g., from 20 up to 30 dB HL or even higher), generating a coherent set of estimates proved challenging due to clinical heterogeneity including variation in the age range, the study setting, the definition of hearing loss and the assessment method. As an example, (i) self-reported hearing loss ranged between 1.0 and 4.0%; (ii) estimates for positive findings measured within speech frequencies in screening studies ranged between 7.4 and 12.8% (using a threshold of > 20 dB HL) and 2.4 and 5.2% (using a threshold of > 30 dB HL), respectively; (iii) whereas estimates based on data collected in registries were between 0.01 and 0.10% for profound hearing loss including deafness or at approximately 4.0% when ICD-10 coding were used. Age- and gender-dependent differences in hearing loss have been reported. Although, our review could not identify such differences in dependence of age, owing to the wide age-ranges included in most the studies, the mean gender ratio males/females was 1.2. Only one study provided estimates on “self-reported” coverage with hearing aids. But valid data on the proportion of hearing impaired children not supplied with hearing aids could not be derived from this study. Likewise, data on the proportion of children and adolescents treated with implantable hearing devices and cochlear implants in the general population are also currently lacking. We identified one other systematic review reporting that hearing loss (derived from screening studies) affected approximately 3.0% of all children and adolescents under 20 years of age living in the United States [31]. Although this estimate is based on lower frequencies than in our review (ranging from 0.5 to 2.0 kHz versus 0.5 to 6.0 kHz in our review), it lies in the range reported in the current review (prevalence range: 2.4 and 5.2%, using a threshold of > 30 dB HL).

### Strengths and limitations of this systematic review

The current systematic review provides prevalence data on hearing loss in the German child and adolescent population. Particularly, it benefits from a thorough and comprehensive literature search including manual searches (e.g. in national registries) to identify data not formally published in an electronic database. Although we considered the risk of bias and assessed representativeness of the available epidemiological data, some issues owing to these assessment methods had to be solved: As an example, there are no well-established tools to assess bias and representativeness of epidemiological studies. Therefore, taking into account published epidemiological literature [14], we developed such criteria both for risk of bias and data representativeness assessment which may also support future systematic reviews in this area.

On the other hand, we are aware that our review findings need to be interpreted with caution due to several limitations: First, risk of bias assessment revealed, for some studies, inappropriate methods to capture prevalence data (e.g. by self-assessment using different questionnaires), which in turn could have led to over- or under-estimation of the true prevalence of hearing loss. The impact of this bias on the reported estimates could, however, not be determined, as none of those studies referring to self-reported data ascertained their prevalence estimates using pure-tone audiograms or another thorough diagnostic assessment. Second, in most studies, external validity (representativeness) of the results was potentially impaired owing to prevalence estimates derived from: (i) children and adolescents living in a special region [19, 26–28]; (ii) data of young patients consulting a physician for a variety of reasons associated with hearing impairments [22–25]; (iii) data provided from selected clinicians [23–25]; or (iv) data lacking currentness (i.e., data referring to the former West Germany) [17, 20, 29, 30]. There would be even older prevalence data available, but we decided not to include such studies because of the very long lapse of time and associated demographical, socio-economical and medical-technical changes since then.

## Conclusions

This systematic review set out to estimate the prevalence of hearing loss in the German population as an example of a Western European country. It also highlights issues associated with estimating the prevalence of hearing loss. Therefore, it has not only implications for pediatricians, general practitioners and otolaryngologists, but also for researchers and those who use epidemiological data to inform political decision making. (i) We found that prevalence data varied broadly reflecting different definitions of hearing loss, different methods of case ascertainment and data collection, different settings and different age-ranges. Generation of a comprehensive and coherent set of prevalence estimates was therefore challenging. *(ii)* Moreover, most of the available data were either not representative for the general population of children and adolescents living in Germany or were considered to be at risk of bias limiting their validity. *(iii)* In view of the negative impact associated with untreated hearing loss, particularly in children and adolescents, as well as the socio-economic costs, a well-done epidemiological study in a more representative population using standardized definitions of hearing loss and objective methods for case ascertainment seems warranted. Such a study would provide reliable age-dependent (national) data on the prevalence of hearing loss, and would allow estimation of the extent of coverage with hearing aids in the general child and adolescent Western European population. (iv) When valid estimates on the prevalence of hearing loss becomes available, one will be better able to address the major preventable causes of hearing loss, develop and disseminate recommendations to prevent them and build partnerships to develop strong hearing care programs including hearing screening and management.

## Supplementary information

**Additional file 1.** Search strategy in Medline. (DOCX 21 kb)

## Data Availability

The datasets used during the current study are available from the first author on request.

## Abbreviations

dB HL: Decibel hearing level
DRKS: Deutsches Register für Klinische Studien, German Register for Clinical Trials
ICD: International Classification of Diseases
kHz: Kilohertz
PRISMA: Preferred Reporting Items for Systematic Review and Meta-Analysis
WHO: World Health Organisation
